# Analysis of the genetic contribution to thoracic aortic aneurysm or dissection in a prospective cohort of patients with familial and sporadic cases in East China

**DOI:** 10.1186/s13023-023-02855-7

**Published:** 2023-08-29

**Authors:** Yanyu Duan, Jianxian Xiong, Zhenghong Lai, Yiming Zhong, Chengnan Tian, Zhiming Du, Zhifang Luo, Junjian Yu, Wentong Li, Weichang Xu, Yabing Wang, Ting Ding, Xuehong Zhong, Mengmeng Pan, Yu Qiu, Xuemei Lan, Taihua Chen, Peijun Li, Kang Liu, Meng Gao, Yanqiu Hu, Ziyou Liu

**Affiliations:** 1 Engineering Research Center of Intelligent Acoustic Signals of Jiangxi Province, Key Laboratory of Prevention and Treatment of Cardiovascular and Cerebrovascular Diseases, Ministry of Education, Gannan Medical University, Ganzhou, 341000 China; 2 Heart Medical Centre, First Affiliated Hospital of Gannan Medical University, Ganzhou, China; 3 Ganzhou Cardiovascular Rare Disease Diagnosis and Treatment Technology Innovation Center, Gannan Medical University, Ganzhou, China

**Keywords:** Thoracic aortic aneurysm or dissection, High-throughput DNA sequencing, TAAD Genetics, Sporadic TAAD

## Abstract

**Background:**

Thoracic aortic aneurysm or dissections (TAADs) represent a group of life-threatening diseases. Genetic aetiology can affect the age of onset, clinical phenotype, and timing of intervention. We conducted a prospective trial to determine the prevalence of pathogenic variants in TAAD patients and to elucidate the traits related to harbouring the pathogenic variants. One hundred and one unrelated TAAD patients underwent genetic sequencing and analysis for 23 TAAD-associated genes using a targeted PCR and next-generation sequencing-based panel.

**Results:**

A total of 47 variants were identified in 52 TAAD patients (51.5%), including 5 pathogenic, 1 likely pathogenic and 41 variants of uncertain significance. The pathogenic or likely pathogenic (P/LP) variants in 4 disease-causing genes were carried by 1 patient with familial and 5 patients with sporadic TAAD (5.9%). In addition to harbouring one variant causing familial TAAD, the *FBN1* gene harboured half of the P/LP variants causing sporadic TAAD. Individuals with an age of onset less than 50 years or normotension had a significantly increased genetic risk.

**Conclusions:**

TAAD patients with a younger age at diagnosis or normotension were more likely to carry a P/LP variant; thus, routine genetic testing will be beneficial to a better prognosis through genetically personalized care prior to acute rupture or dissection.

**Supplementary Information:**

The online version contains supplementary material available at 10.1186/s13023-023-02855-7.

## Background

Thoracic aortic diseases that progress to dissection or rupture manifest a clinical disastrous course with unacceptably high morbidity and mortality [1]. Approximately 21% of patients presenting with aortic dissection die suddenly before seeking medical care [2], and 51% pass away in or en route to the facility to which they are transferred [3]. Acute surgery is the only option for in-hospital patients with type A aortic dissection; however, its overall mortality rate ranges from 8 to 24% [4]. Compared to acute treatment, thoracic aorta dissection repair has a less than 3% perioperative mortality rate and an over 85% 5-year survival rate [5] [6]. Therefore, diagnosis and treatment prior to acute rupture or dissection are likely to have better clinical outcomes.

Currently, the American and European guidelines recommend that the threshold for considering surgical intervention be a thoracic ascending aortic diameter of 5.5 cm [7, 8]. However, acute aortic events occur in more than half of patients who do not meet the criteria for prophylactic surgery, as widely reported [9] [10]. In response to this, a lower bound less than 5.0 cm was given for the timing of surgery for syndromic disorders such as Marfan syndrome (MFS), Loeys‒Dietz syndrome (LDS) and Ehlers‒Danlos syndrome (EDS) [11]. However, some individuals with nonsyndromic familial thoracic aortic aneurysm and dissections (ns-FTAADs) with manifestations restricted to the aorta are also known to have a high risk of dissection at diameters less than 5.0 cm [12, 13]. Therefore, genetic indicators might be required to choose the optimal timing for surgical repair to avoid acute aortic events.

Genetic studies over the past two decades have identified that both ns-FTAADs and syndromic thoracic aortic aneurysm or dissections (s-TAADs) are predominantly single-gene hereditary disorders inherited in an autosomal dominant manner [12]. Syndromic TAADs are caused by pathogenic variants in genes associated with the dysfunction of the extracellular matrix and TGF-β signalling and include Marfan syndrome (*FBN1*), Loeys‒Dietz syndrome (*TGFBR1, TGFBR2, SMAD2, SMAD3, TGFB2, TGFB3*), Ehlers‒Danlos syndrome (*COL1A1, COL1A2, COL3A1, COL5A1 and COL5A2*), arterial tortuosity syndrome (*SLC2A10*), Shprintzen-Goldberg syndrome (*SKI*) and cutis laxa (*EFEMP2, ELN*) [12] [14]. In comparison, the majority of ns-FTAADs arise from pathogenic variants in genes with arterial functionality. Five causative genes were identified in TAAD families: *ACTA2, MYH11, MYLK* and *PRKG1* encode components of the contractile apparatus in vascular smooth muscle cells, and *LOX* encodes an extracellular matrix cross-linking enzyme [15–18]. In addition, the ns-FTAADs of some families can be explained by variants in s-TAAD causative genes, such as *FBN1, TGFBR1, TGFBR2, SMAD3, and TGFB2* [11]. Genetic testing for mutations in these genes can thus contribute to the making of a definitive diagnosis, the choice of the timing of prophylactic surgery, and the optimization of clinical management.

Herein, we analysed 23 known causative genes of TAAD in a cohort of 101 individuals from East China using multiplex PCR targeted amplicon enrichment and deep-coverage massively parallel DNA sequencing, providing insight into the prevalence and clinical characteristics of causative gene carriers.

## Results

### Characteristics of patients

For this study, 101 consecutive patients who presented with thoracic aortic diseases, including 7 thoracic aortic aneurysms (6.8%) and 94 acute aortic syndromes (AASs; 93.1%), were enrolled; the AASs of these patients consisted of 89 aortic dissections, 2 intramural haematomas and 3 penetrating aortic ulcers (Table 1). Approximately 63.7% of patients with thoracic aortic dissections (TADs) had concurrent thoracic aortic aneurysms (TAAs). According to the Stanford classification, 60 out of 89 cases involved the ascending aorta (type A), and the remaining cases involved the descending aorta (type B). Risk factors for TAAD were investigated, including sex, blood pressure and family history (Table 1). Males composed 81.2% (82) of the cohort. Seventy-three patients (72.3%) had hypertension; 78.6% of them were males. Only one patient had a positive family history of an aortic disease, bicuspid valve or coronary artery disease.

**Table 1:** Baseline characteristics of the cohort (n = 101)

Clinical characteristics of the cohort	Mean ± SD or No.(%) patients
Age (years)	57 ± 14 [13–80]
< 50 years at TAAD onset	29(28.7)
≥ 50 years at TAAD onset	72(71.3)
Sex	
Male	82(81.2)
Female	19(18.8)
Thoracic aortic disease	
Aortic aneurysm	7(6.9)
Acute aortic syndrome	94(93.1)
Aortic dissection	91(88.1)
Stanford Type A	60(59.4)
Stanford Type B	29(28.7)
Aortic dissection and aneurysm	58(57.4)
Intramural haematoma	2(2.0)
Penetrating aortic ulcer	3(3.0)
Suspected Syndrome	2(0.2)
Aortic diameter	40.3(7.7)
Z score ≥ 2	46(45.5)
-2 < Z score < 2	49(48.5)
Z score≤-2	2(0.02)
Risk factors and comorbidities:	
Proven positive family history of aortic disease	1(0.1)
Marfan syndrome	2(0.2)
Hypertension	73(72.3)
Smoke history	32(31.7)
Hyperlipidaemia	49(48.5)
Diabetes	51(49.5)
Bicuspid valve	1(0.1)
Coronary artery disease	1(0.1)
Hypothyroidism	0(0.0)
Gastroesophageal reflux disease	0(0.0)
Chronic obstructive pulmonary disease	0(0.0)

### Variants identified by IMPATT assay

**Fig. 1:**
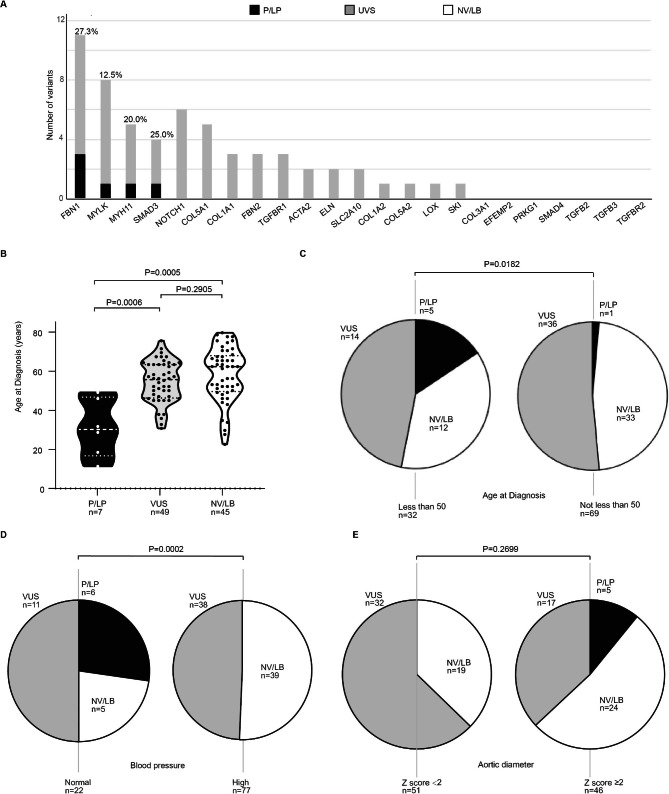
Summary of sequencing results. (A) Numbers of all variants for each gene and percentages of P/LP variants for each gene in the cohort are shown. Impact of age at diagnosis (B, C), blood pressure (D) and arotic diameter (E) on variant category in the whole cohort. Abbreviations: P/LP - pathogenic or likely pathogenic; VUS - variant of uncertain significance; NV/LB – no variant or likely benign

The study revealed that 79 out of 101 patients (78.2%) carried missense variants (Table 2). Fifty-six patients (55.4%) harboured missense variants with minor allele frequency (MAF) thresholds less than 0.1% worldwide (Exac) or in East Asia (Exac). Of them, 6 patients carried more than 1 variant, and 17 patients shared 4 variants (Tables S3 and S4). Thus, the total number of analysed variants in the whole cohort was 69, including 5 pathogenic variants, 1 likely pathogenic variant and 63 variants of uncertain significance (VUS) (Table 3). Sixteen of 23 targeted genes harboured alterations, of which the largest number of VUS and P/LP variants were in the *FBN1* gene, and the highest overall proportion of P/LP variants per gene was also in the *FBN1* gene (27.3%) (Fig. 1A).

**Table 2:** Summary of the variants harboured by TAAD patients

Variable	No. Patients	Percentage
Negative for missense variants	22	21.8%
Positive for missense variants	79	78.2%
Number of variants identified in total:		
Variant related to genetic code		
Missense SNV (no. of variants)	71	
Frameshift (no. of variants)	2	
Nonsense (no. of variants)	2	
Variant related to polymorphism type		
SNP (no. of variants)	71	
Insertion (no. of variants)	1	
Deletion (no. of variants)	5	

Note: No. is the abbreviation for number, and TAAD is the abbreviation for thoracic aortic aneurysm and dissection

**Table 3:** Summary of patients with the variants

101 individuals with	
Variants identified with an MAF > 0.1%	23
Variants identified with an MAF < 0.1%	56
Pathogenic	5
*FBN1*	3
*MYLK*	1
*SMAD3*	1
Likely pathogenic	1
*MYH11*	1
Uncertain significance	49
*ACTA2*	2
*COL1A1*	2
*COL1A2*	1
*COL5A1*	5
*COL5A2*	1
*ELN*	2
*FBN1*	14
*FBN2*	3
*MYH11*	7
*MYLK*	8
*NOTCH1*	5
*SKI*	3
*SLC1A10*	2
*SMAD3*	6
*TGFBR1*	*3*

Six variants were considered to be P/LP: 3 variants in the *FBN1* gene and 1 in each of the *MYH11*, *MYLK*, and *SMAD3* genes (Table 4). Except for two in *FBN1*, the P/LP variants were associated with only isolated aortic disorders. Four VUS were repeatedly identified in TAAD patients: *FBN1 c.1217T > A* (p. Leu406His) in 8 patients (6 Type A, 2 Type B), *SMAD3* c.1180T > A (p. Cys394Ser) in 4 (Type A), *MYH11 c. 1729 A > C* (p. Lys577Gln) in 3 (Type A), and *SKI c.2189 C > T* (p. Pro730Leu) in 2 (1 Type A, 1 Type B) (Table S3); in sharp contrast, these variants had never been found in healthy Chinese individuals [19]. According to protein function prediction from Provean, SIFT, PolyPhen2 and MutationTaster, all variants had a deleterious effect on protein function except for *FBN1 c.1217T > A* (p.Leu406His). Despite this, *FBN1 c.1217T > A* might be associated with TAADs if considering the median age at diagnosis of 48.5 years, which was significantly less than the 57 years in the no variant (NV) group (*P* = 0.0273).

**Table 4:** Pathogenic or likely pathogenic variants identified by the IMPATT assay

No.	Age, Sex	Gene affected	Variant	MAF in Exac		Evidence-based ACMG	Aortic pathology	Clinical diagnosis	Classification	Previously reported?	Family history
				Worldwide	East Asian						
TAAD_6	13,M	*FBN1*	c.114delA p.Ala39fs	0.0000%	0.0000%	PVS	Aortic root aneurysm	Marfan	Pathologic	No	No
TAAD_81	20,M	*FBN1*	c.1285 C > T p.Arg429*	0.0000%	0.0000%	PVS	Aortic root aneurysm	Marfan	Pathologic	No	Yes
TAAD_89	50,M	*MYLK*	c.2736delG p.Lys913fs	0.0000%	0.0000%	PVS	Ascending aortic aneurysm	N/A	Pathologic	No	No
TAAD_90	47,M	*SMAD3*	c.139 C > T p.Gln47*	0.0000%	0.0000%	PVS	Ascending aortic aneurysm	N/A	Pathologic	No	No
TAAD_98	33,M	*MYH11*	c.3787_3789delAAG p.Lys1263del	0.0006%	0.0012%	PS3 + PM4 + PP1	Type A aortic dissection	N/A	Likely Pathologic^1^	No	No
TAAD_102	30,M	*FBN1*	c.7988G > C p.Cys2663Ser	0.0000%	0.0000%	PS1 + PM2 + PP2 + PP3	Type A aortic dissection	Marfan	Pathologic	No	No

Note:

^**1**^ The variant *MYH11 c.3787_3789delAAG* p. Lys1263del was categorized as likely pathogenic for the following reasons: (i) The variant had been proven to decrease the MYH11 level by RT‒qPCR and Western blot (PS3 evidence; we downgraded PM3 into PS3 because the report failed to directly confirm the harmful effect of protein function (PMID: 28,074,631)). (ii) The variant resulted in a change in the length of MYH11 (PM4 evidence). (iii) The variant and TAAD were cosegregated in several family members (PMID: 22,968,129, 26,056,961) (PP1 evidence). (iv) In the ClinVar database, vcv000180420.16 described the variant as likely pathogenic in 1 and a variant of uncertain significance in 7 among 8 submitters, In addition, this variant has been reported in several unrelated individuals affected with thoracic aortic aneurysm and aortic dissection (PMID: 25,907,466, 28,074,631, 28,391,405, 29,510,914, 30,675,029), as well as in two individuals referred for aortopathy genetic testing (PMID: 25,944,730). Finally (v) the protein level was found to be relatively decreased in the surgical specimen (data not shown)

### Genotype-phenotype correlation with P/LP variants

To determine which TAAD patients could benefit from genetic testing, we took the clinical characteristics of the P/LP carriers into consideration (Fig. 1B and E). Noteworthy, a P/LP variant was more likely to be identified in younger patients (Fig. 1B). The median age at diagnosis was 31.5 years for P/LP carriers, 57 years for VUS carriers (*P* = 0.0006) and 57 years in the NV group (*P* = 0.0005). A P/LP alteration was more likely to be found in the patients under the age of 50 years (5 of 32) than in those 50 years or older (1 of 69) (*P* = 0.0182) (Fig. 1C). In addition to the age of onset, three cardiovascular risk factors were analysed for the probability of identifying a P/PL alteration among TAAD patients. Genetic alterations contributed to a much larger percentage of TAAD cases in normotensive patients (6 of 22) than in those with hypertension (0 of 77) (*P* = 0.0002, Fig. 1D). However, the probability of detecting P/LP variants was not associated with normal blood lipid and glucose levels. In addition, several traits of aortic pathology were considered, including the aortic diameter, presence of an aneurysm, and dissection location. We did not find that the likelihood of carrying a P/LP variant was associated with Z scores of a aortic diameter greater than 2 (Fig. 1E), the presence of an aneurysm or Stanford Type A classification. Moreover, no significant difference was found in the percentage of P/LP variants according to the sex of the patients.

Except for the possibility of harbouring a P/LP variant based on those traits, the relative risk was also calculated as described in Table 5. Overall, our results suggested that genetic testing should be performed in TAAD patients who met either of two conditions: an age of onset less than 50 years and normotension. Additionally, when a patient is identified as carrying a pathogenic variant, genetic testing should be recommended for his or her blood relatives.

**Table 5:** Relative risk of carrying a pathogenic or likely pathogenic variant according to diverse phenotypes

	Total	P/LP (percentage)	RR (95% CI)	P value
Age-of-onset, < 50	32	6 (18.8)	13.1250 (1.6474-104.5677)	0.0150
Female	19	0 (0.0)	0.3192 (0.0187–5.4353)	0.4298
Normotension	22	6 (27.3)	44.0870 (2.5793-753.5646)	0.0089
Normolipidaemia	30	2 (6.7)	7.5758 (0.3755-152.8386)	0.1865
Normoglycaemia	36	2 (5.6)	2.8333 (0.2669–30.0762)	0.1256
Z scores of aortic diameter, >2	46	5 (10.9)	5.5435 (0.6722–45.7129)	0.1116
Presence of aortic aneurysm	66	5 (7.6)	1.3258 (0.2709–6.4870)	0.7278
Stanford Type A	60	3(5.0)	0.7750 (0.1366–4.3974)	0.7750

## Discussion

The present study was a one-year prospective trial aimed at determining the prevalence of genetic abnormalities in thoracic aortic disease according to the data of 101 consecutively enrolled unrelated patients from the Department of Cardiovascular Surgery. Since the cost was free, all patients in whom the IMPATT assay was recommended were able to undergo genetic testing; subsequently, the total frequency of P/LP variants among 23 known TAAD-associated genes was found to be 5.9%. This frequency is similar to the 3.9% and 4.9% reported previously, respectively, in a whole-exome sequencing study of 102 TAAD patients [20] and in a large cohort study of 1025 TAAD patients [21]. Even though we met the most stringent criteria of the ClinGen framework, including only 9 definitive causes[22], the percentage of P/LP variants was still 5.9%, close to that of the above two studies [20, 21]. A mixed cohort of sporadic and familial cases in South China had been previously reported to have a total frequency of P/LP variants of 22.5% (34/151) [23], which was far greater than the prevalence of P/LP variants in our study and the two other studies as described above [21] [20]. However, given that the P/LP variants were identified from 129 candidate genes with little evidence reported for being TAAD-causing variants in the majority, the total frequency could remain enormously overestimated in South China [22]. In addition, we repeatedly identified some VUS in TAAD patients but never in local healthy people, indicating the importance of genetic factors in thoracic aortic disease.

The *FBN1* gene was the greatest contributor to TAAD cases in patients harbouring 3 out of 6 P/LP variants. Three carriers of a P/LP variant in *FBN1* met the clinical criteria for a diagnosis of MFS according to the 2010 revised Ghent nosology criteria [24], of whom two displayed typical MFS manifestations involving multiple organ systems and one manifested signs isolated strictly to the ascending aorta with the dilatation of the aortic root. Notably, one of two patients with typical MFS carried a de novo variant that was not present in his parents or four siblings [25]. Consistent with our observation, *FBN1* pathogenic variants have been demonstrated to be associated with a wide range of phenotypic variabilities from single organ involvement to a multiorgan dysfunction syndrome; the dysfunction of *FBN1* is a common pathogenesis of aortic disease in MFS, FTAADs and sporadic TAADs[26] [27] [28]. The type and location of pathogenic *FBN1* variants affect TAAD progression [27], consequently providing some insight into the prognostic stratification of TAAD cases based on the variants. Moreover, it is very likely that a variant in *FBN1* arises spontaneously, sometimes without full penetrance, and involves different organs among carriers of the same variant [25] [29], indicating the difficulty of family history tracking.

As an important risk factor for TAAD [30], genetic testing is required to identify pathogenic variants in patients. Studying the clinical features of high-risk individuals harbouring a variant prone to dissection would be beneficial to improve the efficiency. Two risk factors were significantly associated with genetic disorders, including an age at diagnosis less than 50 years and normotension (Table 5). The median age of onset of P/LP carriers was much lower than that of the rest of the cohort, consistent with an observation in a large cohort [21]. Compared with the age at diagnosis in patients with familial (56.8 years) and sporadic (64.3 years) cases, the lower age of onset identified in P/LP carriers again proved that genetic defects accelerated TAAD progression[21]. Correspondingly, in this study, genetic defects were 13.1 times (95% CI: 1.6474-104.5677, *P* = 0.0150) more likely to be identified in the patients with an age of onset before 50 years than in those with an age of onset after 50 years.

Hypertension was strongly associated with incident thoracic aortic dissection [30]. The wall tension of the aorta is directly proportional to blood pressure, thus explaining why hypertension is a major risk factor for TAAD [31]. Hypertension accelerates the progression of the usual histopathologic changes in the aorta associated with TAAD [7]. In our study, in TAAD patients with normal circumferential stress on the aorta, the possibility of a vascular inborn error was over 44.1 times (2.579-753.565, *P* = 0.0089) higher than in those with hypertension.

Although the association of aortic root enlargement with aortic dissection seems straightforward, we found no significant association between variant categories and three aortic traits, including Z scores of the aortic diameter, presence of aneurysm, and dissection location. It has been proven that some pathogenic variants in genes such as *MYLK* [32] are not always preceded by obvious aortic dilatation. Moreover, arterial hypertension, as a predisposing condition for the development of thoracic aorta aneurysms, further makes the association more complex [7].

Our study is subject to a number of limitations. First, the number of P/LP variants might be underestimated if stringent criteria on variant classification in the guidelines of the American College of Medical Genetics and Genomics (ACMG) are considered. Several VUS carried by more than 1 patient in this study were not present in population databases, yet some of them were classified as VUS associated with ns-FTAAD in previous reports. Although extremely rare variants in causative genes are often disease related, diagnostic uncertainty is apparent solely based on the genetic technology itself, indicating the need for high-throughput functional assays to systematically classify genetic alterations [33] [34]. Moreover, our IMPATT assay specifically analysed the coding sequences and intron/exon boundaries of 23 TAAD-related genes (Table S1) but neglected noncoding variants despite their known small effects on disease; subsequently, genome-wide sequencing could enrich our understanding of TAAD-related genetic factors. Furthermore, we were unable to track the cause of death, and thus accurately track the family history of aortic disease, for the deceased parents of the patients due to limited local medical resources before 2000. Future studies will aim to correlate VUS carriers’ family histories based on further follow-up.

## Conclusions

The study identified that the frequency of genetic variants causing TAAD was 5.9% among 101 affected patients in this prospective cohort. Positive carriers had nonsyndromic manifestations of TAAD except for two patients who had typical manifestations of MFS. Two clinical features, age under 50 years at diagnosis and normotension, were found to increase the likelihood of harbouring P/LP variants. If the potential for de novo variants and unknown family history are taken into consideration, the high probability of a P/LP variant among these individuals suggests that routine genetic screening is still worth recommending.

## Methods

### Ethical compliance

All procedures performed in this study involving human participants were in accordance with the guidelines outlined in the Declaration of Helsinki (as revised in 2013).

### Patient profile

Patients who were diagnosed with TAAD by cardiovascular surgeons at the Heart Medical Centre of the First Affiliated Hospital of Gannan Medical University, Jiangxi, China, between August 2020 and August 2021 were included in this study (clinical trial registration no. ChiCTR2000034841 [clinicaltrials.gov]). Patients were included if he or she had TAAD and were aged no more than 80 years and were excluded when one of the following existed: pseudoaneurysm, heart surgery history, bone marrow transplantation, exogenous transfusion in the past six months, and pregnancy. Peripheral blood samples were collected from all participants before surgery. MFS diagnoses were made according to the 2010 revised Ghent nosology criteria [24]. Family history inquiry showed that no kinship was found in any reported individuals.

### Targeted exon sequencing

The alterations in the known TAAD-associated genes were profiled using our IMPATT assay (Integrated Mutation Profiling of Actionable TAAD Targets), which utilizes multiplex PCR targeted amplicon enrichment and deep-coverage massively parallel DNA sequencing. The coordinates of 23 targeted genes in Table S1 were determined using the human reference genome (NCBI GRCh37) [35]. Custom primers for the multiplexed PCR approach were designed to enrich 754 amplicons covering all protein-coding exons plus 15 bp adjacent intron padding of the targeted genes using Illumina’s DesignStudio Sequencing Assay Designer. The total targeted DNA length was 80.86 kb. The average amplicon length was 123 bp with 48% GC content. The library was prepared using two primer pools for multiplex PCR, indexed using index adapters, further amplified, and combined in preparation for PE150 sequencing in the HiSeq XTen platform as described in the manufacturer’s AmpliSeq for Illumina workflow (Illumina, San Diego, CA).

### Read mapping, variant calling & annotation

The sequencing read quality was assessed using FASTQC. Primer sequences were trimmed from FASTQ files using cutPrimers [36] prior to read mapping to the human GRCh37/hg reference sequence using the Burrows‒Wheeler Aligner V0.5.17 [37]. After the removal of potential PCR duplicates (Picard, http://picard.sourceforge.net) and realignment (GATK) [38], mutations were called and filtered using SAMtools [39], GATK3.7 [38], Pindel [40], BreakDancer [41] and CNVnator [42]. All variants were annotated according to the control population of the 1000 Genomes Project (2014 October release) [43], ExAC r0.3.1 [44], EVS (https://evs.gs.washington.edu/EVS/), the disease databases of ClinVar [45], and OMIM [46].

### Variant and sample filtering

Synonymous variants and intronic variants outside splice sites were not considered for downstream analysis only when they were annotated as pathogenic or disease-causing in ClinVar [45] or HMGD [47]. Variants with MAF thresholds greater than 0.1% in either the worldwide superpopulation or the East Asian population ExAC r0.3.1 [44] datasets were excluded. Samples with less than 80% of target bases covered by more than 60 reads were excluded from downstream analysis.

### Pathogenicity assignment

Variants passing filters were assigned to one of four categories, ‘pathogenic’, ‘likely pathogenic’, ‘VUS’ or ‘likely benign’, based on ACMG criteria [48].

In particular, a variant was considered a pathogenic variant if it (I) had been reported as disease causing in HGMD or pathogenic in ClinVar, (II) resulted in the same amino acid substitution as an HGMD disease-causing variant or ClinVar pathogenic variant, (III) was a de novo variant if nonsense (i.e., had an in-frame and out-of-frame deletion/insertion, the splice site variants affected the canonical splice sequence, or was shown to alter splicing on an mRNA level), (IV) was missense and either affected/created cysteine residues or affected conserved residues of the EGF consensus sequence in *FBN1* [49], (V) had a substitution of a glycine residue within a GlyXY repeat in collagen triple helical domain, (VI) had an insertion of amino acids disrupting the GlyXY repeat sequence in collagens, or (VII) had an alteration of a key residue in a protein feature in keeping with previously ascribed molecular mechanisms for a given gene [21]. The pathogenic or likely pathogenic (P/LP) variants and four VUS were confirmed by Sanger sequencing (Table S2).

### Statistical analysis

Fisher’s exact test was used for the assessment of categorical variables between different groups. Nonparametric phenotypic continuous measurements were analysed using the unpaired Wilcoxon rank-sum test. *P* values less than 0.05 were considered statistically significant (two-sided). All calculations were performed using SPSS 24.0 software.

### Electronic supplementary material

Below is the link to the electronic supplementary material.

Supplementary Material 1. Additional file 1. Table S1. List of 23 targeted genes for multiplex PCR-targeted amplicon enrichment. Additional file 2. Table S2. Primer sequences for Sanger validation of 9 pathogenic or likely pathogenic and 4 shared genetic variants. Additional file 3. Table S3. Four variants of uncertain significance were shared in 17 patients. Additional file 4. Table S4. Six patients had more than 1 variant of uncertain significance

Supplementary Material 2

## Data Availability

The datasets used and/or analysed during the current study are available from the corresponding author on reasonable request.

## List of abbreviations

AAS: acute aortic syndromes
EDS: Ehlers-Danlos Syndrome
ns-FTAAD: non-syndromic familial thoracic aortic aneurysm and dissection
MFS: Marfan Syndrome
LDS: Loeys-Dietz Syndrome
NV: no variation
P/LP: pathogenic or likely pathogenic
s-TAAD: syndromic thoracic aortic aneurysm or dissection
TAA: thoracic aortic aneurysm
TAAD: thoracic aortic aneurysm or dissection
VUS: variant of uncertain significance
